# Pyruvate dehydrogenase kinase 4 exhibits a novel role in the activation of mutant KRAS, regulating cell growth in lung and colorectal tumour cells

**DOI:** 10.1038/onc.2017.224

**Published:** 2017-07-10

**Authors:** A G Trinidad, N Whalley, R Rowlinson, O Delpuech, P Dudley, C Rooney, S E Critchlow

**Affiliations:** 1 AstraZeneca iMED Oncology, Alderley Park, Macclesfield, Cheshire, UK; 2 AstraZeneca iMED Oncology, Cambridge, UK

## Abstract

RAS signalling is involved in the control of several metabolic pathways including glycolysis, mitochondrial respiration and glutamine metabolism. Importantly, we have found here that loss of PDHK4, a key regulator of the pyruvate dehydrogenase complex, caused a profound cell growth inhibition in tumour cells harbouring KRAS mutations. Using isogenic cells and a panel of colorectal and lung cell lines we demonstrated that KRAS mutant cells showed a dependency on PDHK4 whereas KRAS wild-type cells were significantly resistant to PDHK4 knockdown. We have found that PDHK4 plays a role in the post-translational regulation of mutant KRAS activity. Depletion of PDHK4 causes disruption of KRAS cellular localization, a reduction in KRAS activity which, in turn, results in reduced MAPK signalling. Interestingly, PDHK4 and KRAS depletion resulted in a similar metabolic phenotype consisting of a reduction of glucose and fatty acid oxidation. Moreover, stable expression of PDHK4 increased localization of activated KRAS at the plasma membrane and induced tumour cell growth *in vitro* and *in vivo*. Taken together these data support a model where PDHK4 regulates KRAS signalling and its tumorigenic properties and suggest that inhibition of PDHK4 could represent a novel therapeutic strategy to target KRAS mutant colorectal and lung cancers.

## Introduction

Cancer cells acquire genetic mutations which can potentially alter signalling pathways dependent on growth factors and nutrient supply. In particular, many of the well-characterized oncogenes, including *PIK3CA*, *AKT*, c*-MYC* and *RAS*, have been shown to promote glycolytic pathways, while tumour suppressors tend to inhibit glycolysis.^1, 2^ The *RAS* genes were the first oncogenes identified and are the most frequently mutated proteins in human cancers. While mutations in KRAS are more frequent in pancreatic, colon and lung carcinomas, HRAS mutations are predominantly found in bladder cancer, and NRAS mutations are associated primarily with hematopoietic malignancies and melanomas.^3^ Each RAS protein is a 21 kDa guanine nucleotide binding protein with an intrinsic GTPase activity which transduces signals by interacting with the effectors only in the guanosine triphosphate (GTP)-bound conformation. RAF1 was established as the first known effector which activates the MAPK-ERK pathway,^4^ but other family of proteins have also been shown to interact with RAS-GTP including PI3-Kinase, RAL-specific GEFs, TIAM1 and PLCepsilon.^5, 6^ In addition to GTP binding, RAS proteins must also be associated with cellular membranes in order to transduce signals, and post-translational modifications are required for the proper trafficking and localization of RAS within the cell.^7^ Recently, a new direction in RAS research has focussed on the link between RAS activation and cancer metabolism. KRAS has been shown to promote glycolysis by increasing expression of glucose transporter 1 (GLUT1).^8^ In addition, KRAS mutant pancreatic tumours use glutamine metabolism and lower intracellular reactive oxygen species for optimal tumour growth.^9^ Other studies have demonstrated that autophagy and mitochondrial reactive oxygen species generation is required for KRAS-induced cell proliferation and tumorigenesis.^10, 11^

The pyruvate dehydrogenase complex (PDC) has a key role in regulating metabolic flux linking the glycolytic pathway and tricarboxylic acid (TCA) cycle. The mammalian PDC complex is composed of three functional enzymes: E1, E2 and E3 organized around a 60-meric dodecahedral core formed by E2 (dihydrolipoyl transacetylase) and the E3-binding protein that bind to E1 (pyruvate dehydrogenase, PDH) and E3 (dihydrolipoamine dehydrogenase). PDH is highly regulated by four different pyruvate dehydrogenase kinase PDHK isoforms (PDHK1, 2, 3 and 4) which differ in tissue expression and regulatory characteristics.^12^ Importantly, therapeutic inhibition of PDHK activity by dichloroacetate has been reported to reverse metabolic remodelling in tumour cells, and promote apoptosis and cause cell growth inhibition in certain cancers *in vivo* including glioblastoma, colon, prostate and metastatic breast tumours.^13, 14^ However, dichloroacetate is a low potency, pan-PDHK inhibitor that requires high doses for its therapeutic effects.^15^ Phosphorylation of PDH at any of the three sites Ser^232^, Ser^293^ and Ser^300^ inhibits its activity, resulting in the inhibition of the glucose oxidation.^16^ Interestingly, PDHK1 has been reported to phosphorylate all three sites, but PDHK2, 3 and 4 display specificity for Ser^293^ and Ser^300^.^17^ While the transcription of PDHK1 and 3 genes is activated by low oxygen levels in response to HIF-1α in tumour cells,^18, 19^ PDHK4 expression is upregulated in tissues with high rates of fatty acid synthesis, suggesting a critical role in lipid metabolism.^20^ The roles of PDHK2 and PDHK4 have been reported to be more relevant in starvation and diabetes, as their expression levels can be controlled by nutritional factors, hormones, steroids and fatty acids.^21^

Here, we show for the first time, that PDHK4 down-regulation significantly inhibits the growth of KRAS mutant tumours, which is uncoupled from PDH regulation. Mechanistic studies demonstrate that this phenotype is correlated with a decrease in active KRAS and disruption of KRAS subcellular localization and MAPK signalling. Consistently, stable expression of PDHK4 enhanced *in vitro* cell growth in 3D cultures and *in vivo* tumour growth. We therefore propose a novel function of PDHK4 in the activation of mutant KRAS in lung and colorectal cancer.

## Results

### KRAS mutant tumour cell lines are sensitive to PDHK4 knockdown

**Figure 1:**
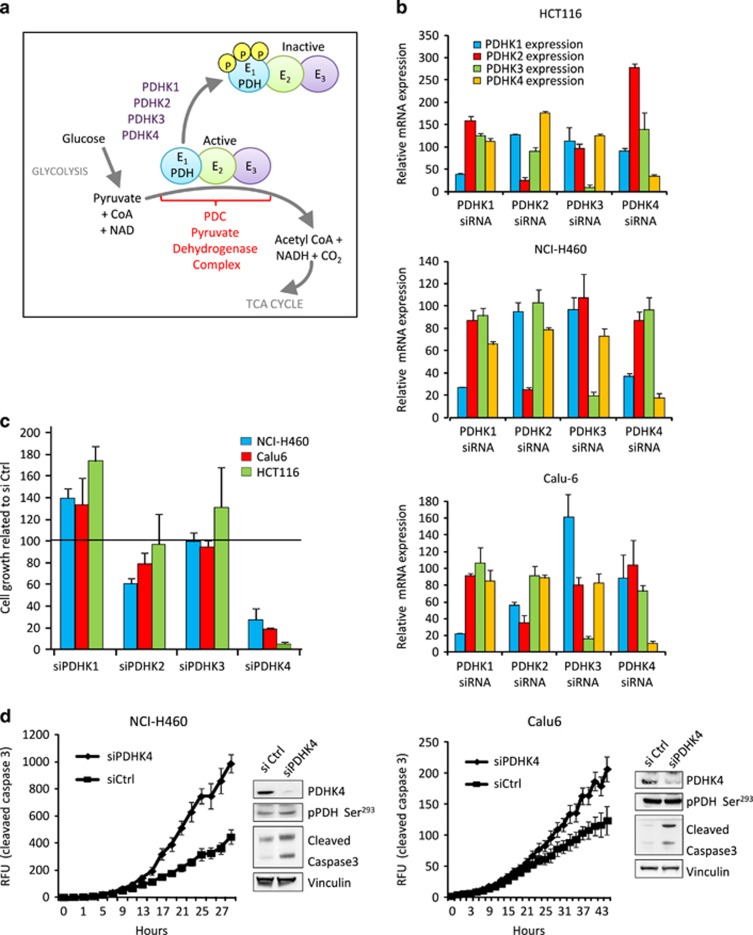
PDHK4 inhibits cell growth in mutant KRAS cells. (**a**) Schematic representation of the PDC complex activation cycle, regulated by its phosphorylation status. (**b**) KRAS mutant cells HCT116, NCI-H460 and Calu-6 were transfected for 48 h with specific siRNA SMART-pools targeting all PDHK isoforms at 20 nM. mRNA levels were verified by qRT–PCR and relative expression was calculated using the delta-delta Ct method normalizing to 18S rRNA and plotted relative to the control. (**c**) Cell growth in mutant KRAS cell lines was analysed by Sytox green cell count assay after 72 h of PDHK-1, 2, 3 and 4 knockdown. Chart shows the percentage of cell growth after knockdown related to the Ctrl siRNA treatment and error bars represent the SEM of replicates (*n*=3). (**d**) Caspase 3 activity was measured in NCI-H460 and Calu-6 cells using the NucView reagent following transfection with PDHK4 siRNA. Cleaved caspase 3 and PDHK4 protein levels were also verified by western blot. Error bars represent the s.e.m of replicates (*n*=3).

The activities of PDHK1, 2, 3 and 4 are enhanced when levels of ATP, NADH and acetyl-CoA are high, resulting in the inhibition of the PDC complex and a promotion of glycolytic phenotype essential for tumorigenesis. Conversely, an increase in pyruvate inhibits PDHKs and activates the pyruvate dehydrogenase phosphatases (Figure 1a). Recently, there has been an interesting report linking PDHK1 and BRAF in melanoma.^22^ It has also been shown that activation PDHK by phosphorylation or by upregulation of gene expression is induced by different oncogenes such as MYC, HIF-1, FGFR1 or BCR-ABL.^23^ Given the importance of oncogenic KRAS in maintaining the glycolytic phenotype in cancer cells, we decided to investigate the role of PDHKs and PDH activation in KRAS mutant cancer models. Following validation of the specific knockdown of PDHK isoforms by siRNA in cells harbouring KRAS mutations (Figure 1b), cell growth assays revealed that all mutant KRAS cells assessed were significantly sensitive to PDHK4 depletion but not to the depletion of the other isoforms (Figure 1c). Moreover, PDHK4 knockdown in mutant KRAS cells was correlated with an increase in apoptosis assessed by measurement of cleaved caspase 3 levels using NucView fluorescent assay, and western blot analysis (Figure 1d). However, no significant effect on PDH phosphorylation at Ser^293^ or Ser^300^ was observed upon PDHK4 knockdown in these cells, indicating that the effect on cell growth was not due to an increase in PDH activity (Figure 1d, Supplementary Figures S1C and D). Together, these results suggest a PDH-independent role of PDHK4 in maintaining cell growth in mutant KRAS tumours.

### Lung and colorectal wild-type KRAS tumour cells are resistant to PDHK4 depletion

**Figure 2:**
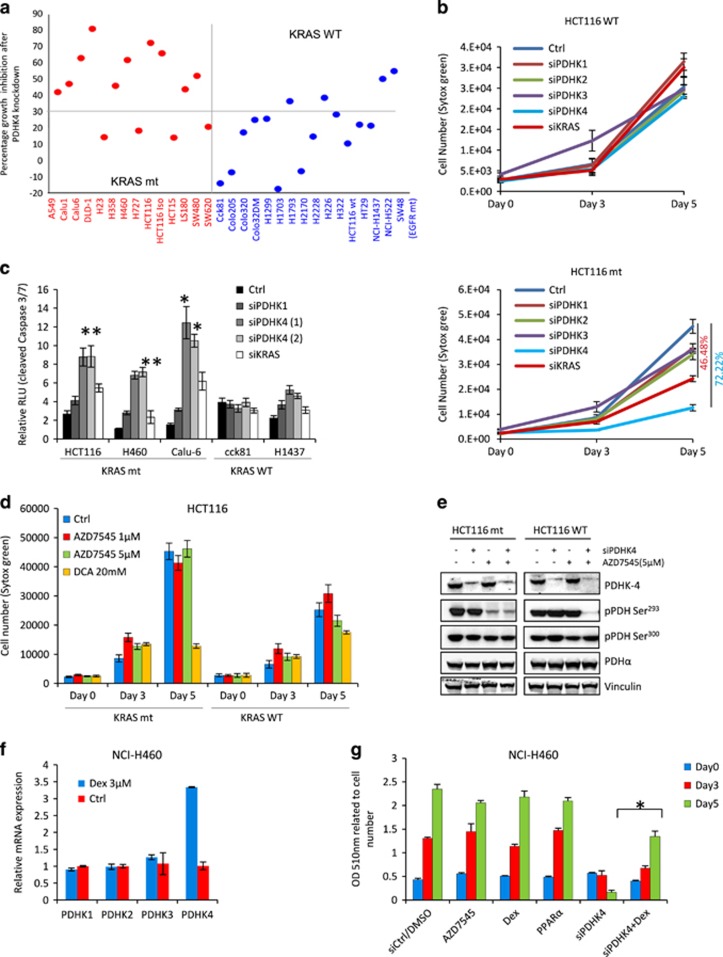
Colorectal and lung wild-type KRAS cells are resistant to the phenotypic effect on cell growth after PDHK4 downregulation. (**a**) PDHK4 knockdown was performed in a panel of wild-type and mutant KRAS cells. Cells were counted 3 days later. The number of PDHK4 siRNA treated cells was compared to the number of cells treated with the control siRNA and percentage of growth inhibition was plotted. (**b**) Isogenic HCT116 cell lines were transfected at the indicated times with siRNAs targeting all PDHK isoforms and KRAS (10 nM). Cell growth was determined by sytox green cell count assay. (**c**) KRAS mutant and WT cells were transfected with siRNA targeting PDHK1, PDHK4 and KRAS as indicated. Caspase 3 and 7 activity was determined by luciferase caspase-Glo assay after 48 h of transfection. (**d**) Isogenic HCT116 cells were treated as indicated with the PDHK inhibitors AZD7545 (1 and 5 μM), and DCA (20 mM) and cell number was assessed by sytox green. (**e**) HCT116 cells were treated with PDHK4 siRNA for 48 h followed by the addition of AZD7545. Cells were collected after 24 h of AZD7545 treatment and western-blots were performed with indicated antibodies. (**f**) NCI-H460 cells were treated with 2 μM of dexamethasone for 5 days and mRNA levels of all PDHK isoforms were detected by qRT–PCR. Relative expression was calculated using the delta-delta *C*_*t*_ method normalizing to 18S rRNA and plotted relative to the control. (**g**) NCI-460 cells were transfected with PDHK4 siRNA for 24 h followed treatment as indicated times with 5 μM of AZD7545, 10 mM of DCA and 2 μM of Dexamethasone. A measurement of cell number was performed by Sulforhodamine B colorimetric assay and cell number was normalized to optical density values at 510 nm. All graphs show the mean±s.e.m from independent experiments (*n*=3). *P*-values were calculated according to *t*-student scores and considered significant with values of *P*<0.05 two-sided paired *t*-test. DCA, Dichloroacetate

We then performed an extended analysis in a large cell panel including several KRAS wild-type and mutant colorectal and lung cell lines. Using a specific siRNA SMART-pool to target PDHK4, we observed an enhanced sensitivity to PDHK4 knockdown in most of mutant KRAS cells assessed compared to wild-type cells (Figure 2a). Some of the wild-type KRAS cell lines such as SW48 (EGFR mutant), H522 and H1563 also displayed certain sensitivity to PDHK4 depletion and some of the mutant were resistant (H23, H727 and SW620), potentially due to alternative biological functions of PDHK4 or differential genetic backgrounds. Overall, the responses to PDHK4 knockdown show an interesting tendency for increased cell growth inhibition in mutant KRAS cells compared to wild-type cells (Figure 2a, Supplementary Figure S1E). Importantly, this differential sensitivity in wild-type and mutant cell lines was not linked to total protein expression levels of KRAS or PDHK4 (Supplementary Figures S1A and B). Moreover, a clear time-dependent cell growth inhibition after PDHK4 siRNA treatment was also observed in the mutant KRAS cells Calu-6 and H460, but not in wild-type KRAS cells HT29 and H1437 (Supplementary Figure S1F). To confirm whether this effect of PDHK4 depletion on cell growth was exclusively dependent on the KRAS mutational status, we investigated the impact of PDHK depletion on cell growth in KRAS isogenic cells. Knockdown of all of the PDHK isoforms and KRAS confirmed a dependence of KRAS mutant cells on PDHK4 and KRAS, which is not evident in KRAS wild-type cells (Figure 2b). However, we cannot compare the degree of phenotypic effect following PDHK4 or KRAS knockdown due to potential differences in protein half life and knockdown levels. Consistent with these data, analysis of cleaved caspase 3/7 levels following PDHK4 depletion indicated a higher level of apoptosis in mutant KRAS cells than in wild-type (Figure 2c).

To further explore this isoform selective dependence of KRAS mutant cells on PDHK4, we used the compound AZD7545, a selective inhibitor of PDHK1 and 2 which has been shown to increase PDHK4 activity.^24^ AZD7545 inhibited growth of KRAS WT cells, but had no effect on the growth of KRAS mutant cells. The pan-PDHK inhibitor dichloroacetate inhibited growth of both mutant and wild-type cells (Figure 2d). To determine if this differential effect was due to PDHK4, we combined AZD7545 and PDHK4 knockdown and measured the phosphorylation status of PDH in the KRAS isogenic cells (Figure 2e). When PDHK4 is knocked down, in either cell type, there is no effect on pPDH Ser^293^ (Figure 2e, Supplementary Figures S1D and S5C). When AZD7545 and PDHK4 siRNA was combined there was a reduction in PDH phosphorylation, in both cell lines. These data suggest that in both KRAS mutant and wild-type cell lines, alternative PDHK isoforms can phosphorylate PDH to compensate for the loss of PDHK4. Combined inhibition/depletion of PDHK1, 2 and 4 had very little effect on pSer^300^. Interestingly, in KRAS wild-type cells inhibition of PDHK1 and 2 (but not PDHK4) with AZD7545 had no effect on PDH phosphorylation, suggesting compensation by PDHK4. In contrast, inhibition of PDHK1 and 2 in KRAS mutant cells lead to a decrease in PDH phosphorylation (Ser^293^), suggesting that in the KRAS mutant setting PDHK4 is unable to compensate for the loss of PDHK1 and 2 activity (Figure 2e, Supplementary Figures S2B and S5C). Consistent with the expected mode of action, inhibition of the phosphorylation of PDH on Ser^293^ by AZD7545 (without the compensation by PDHK4) in KRAS mutant cells led to an increase in overall oxygen consumption rate (OCR) (Supplementary Figure S2A), without affecting cell growth. Taken together, these data suggest that the cell growth inhibition seen following PDHK4 depletion in KRAS mutant cells is via a mechanism that is independent of PDH activity.

Finally, to confirm the reliance of mutant cells on PDHK4 for growth, we tested whether a specific PDHK4 transcriptional activator such as Dexamethasone^25^ would revert the cell growth phenotype caused by PDHK4 knockdown. We first confirmed that dexamethasone treatment specifically increased PDHK4 mRNA levels in the KRAS mutant NCI-H460 cells (Figure 2f). Importantly, PDHK4 knockdown following 4-day pre-treatment with dexamethasone resulted in a partial rescue of cell growth (Figure 2g). Collectively, these findings suggest that PDHK4 is necessary for tumour growth in mutant KRAS cells, which appears to be uncoupled from the action of PDHK4 on the PDC complex.

### PDHK4 knockdown reduces mutant KRAS protein expression and shows a modest transcriptional suppression of *MEK*-target genes compared to wild-type KRAS cells

**Figure 3:**
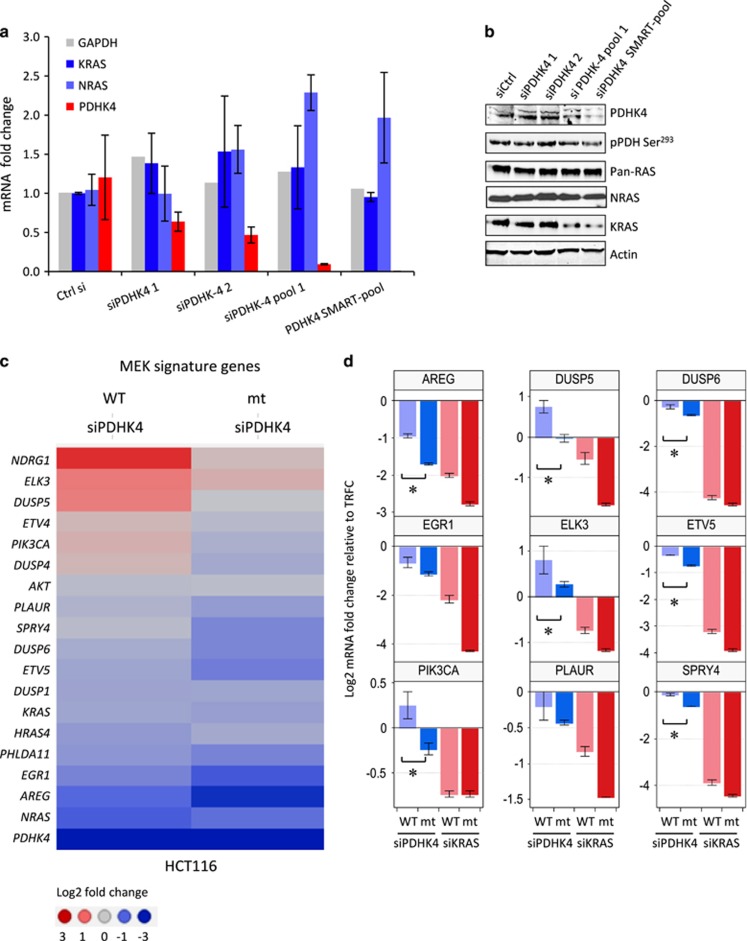
PDHK4 knockdown reduces mutant KRAS protein expression and shows a modest transcriptional suppression of MEK-target genes compared to wild-type KRAS cells. Knockdown of PDHK4 after 72 h was validated in KRAS mutant HCT116 cells with 10 nM of different specific single oligos (siPDHK4 1 and 2) and siRNA pool 1 and SMART-pool as indicated. (**a**) mRNA expression of PDHK4, glyceraldehyde 3-phosphate dehydrogenase, KRAS and NRAS was analysed by qRT–PCR represented as relative Ct values and (**b**) protein levels were tested by immunoblot using specific antibodies for PDHK4, pPDH Ser^293^, Pan-RAS, NRAS and Actin. (**c**) Gene expression profiling using Fluidigm dynamic array in isogenic HCT116 cells after 48 h of KRAS and PDHK4 knockdown (SMART-pool). Heatmap corresponds to a subset of MEK signature genes and represents the log2 fold change *C*_*t*_ values as indicated (3 to −3). (**d**) Statistical analysis of a subset of some of these MEK signature genes represents the changes in gene expression as log2 fold change upon PDHK4 and KRAS downregulation. Bars correspond to the±s.e.m of replicates (*n*=3) and *P*<0.05 two-sided paired *t*-test.

To investigate whether the phenotypic effects in mutant KRAS cells after PDHK4 knockdown were due to changes in the mRNA and/or protein expression of N- and KRAS, we first validated single and pooled PDHK4 siRNA oligos in mutant KRAS HCT116 cells. Single siRNA oligos only partially reduced PDHK4 mRNA levels without affecting N- and KRAS mRNA expression levels (Figure 3a). Both PDHK4 siRNA pool 1 (combination of two single oligos) and siRNA pool 2 (SMART-pool used previously) achieved significant PDHK4 mRNA and protein downregulation, which, interestingly, was correlated with a reduction in KRAS protein but not mRNA levels (Figure 3b). To further extend the analysis of the transcriptional effect following PDHK4 siRNA treatment with the validated SMART-pool, we used targeted fluidigm transcript profiling to measure the expression of PDHK4, the RAS isoforms and a set of genes known to be modulated by MEK^26^ in the isogenic HCT116 cells (Supplementary Figure S3B). PDHK4 siRNA treatment had no impact on KRAS gene expression in wild-type or mutant cells (Supplementary Figure S3A). However, PDHK4 downregulation caused a significant impact on some, but not all MEK signature genes in mutant KRAS cells (Figure 3c). A statistically significant reduction of *AREG, DUSP5, DUSP6, ELK3, ETV5, PI3KCA* and *SPRY4* was observed under PDHK4 depletion in mutant cells compared to wild type. These genes have been shown to be critical downstream effectors of the KRAS signalling pathway (Figure 3d).^26^ These data suggest that PDHK4 depletion suppresses mutant KRAS protein expression levels by a post-translational mechanism, leading to a modest reduction of some *MEK* signature genes.

### PDHK4 is required to maintain KRAS signalling pathway activity and cellular localization

**Figure 4:**
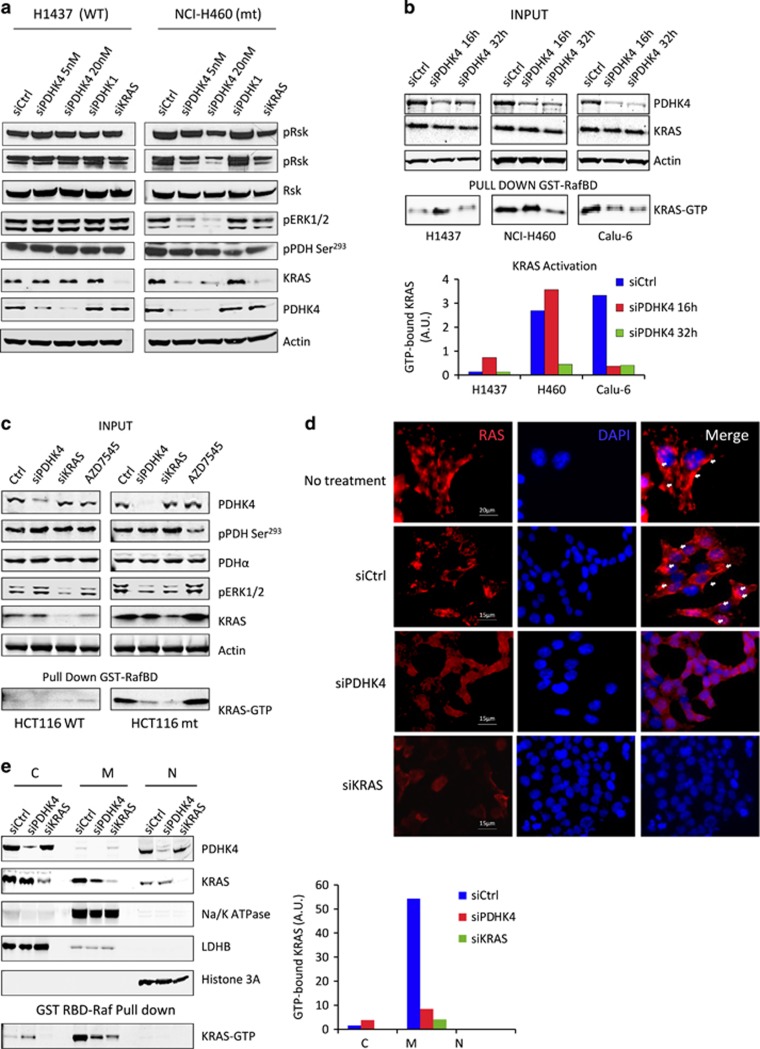
PDHK4 is essential to maintain KRAS signalling pathway, activity and cellular localization. (**a**) NCI-H1437 and NCI-H460 cells were treated with PDHK4 (5 and 20 nM), PDHK1 (10 nM) and KRAS (10 nM) siRNA for 72 h and RAS signalling pathway activation was assessed by western blotting using phospho-specific antibodies as indicated. (**b**) RAS activity was measured using RAF1-RBD-RAS GTP-specific pull-down assay using GST-tag beads after PDHK4 knockdown treatment at 16 and 32 h. Graph represents the quantification of GTP-bound KRAS pool related to KRAS total expression levels. (**c**) Isogenic HCT116 cells were treated for 48 h with PDHK4 and KRAS siRNAs (10 nM) and 5 μM of AZD7545 (PDHK1/2 inhibitor). Lysates were subjected to the same KRAS GTPase-specific pull-down assays in (**b**) and total proteins were detected by the indicated antibodies following western blot analysis. (**d**) HCT116 cells were fixed in paraformaldehyde 4%, blocked and stained with Pan-RAS antibody (Red) and nuclear counter-stain 4',6-diamidino-2-phenylindole (blue) after 48 h of PDHK4 and KRAS knockdown (10 nM siRNA). White arrows correspond to specific compartment localization of RAS staining. (**e**) Cell fractionation was performed in HCT116 cells after 48 h of PDHK4 and KRAS knockdown. Each fraction was subjected to immuno-blotting and KRAS GTPase-specific pull-down assay. Chart represents the quantification of GTP-bound KRAS in the cytoplasmic, membrane and nuclear fractions normalized to LDHB, Na/K ATPase and Histone 3A levels respectively.

To validate that the effect of PDHK4 depletion on KRAS protein expression was not a result of an off-target siRNA effect, we expressed a doxycycline-inducible PDHK4 shRNA in the HCT116 isogenic model. Treatment with 2 μg/ml doxycycline for 3 days resulted in more than 85% of protein knockdown in both wild-type and mutant KRAS HCT116 cells (Supplementary Figure S4A). Induction of PDHK4 shRNA also elicited a similar cell growth inhibition and reduction in KRAS protein expression levels in mutant KRAS cells, but not in wild-type, as seen previously with the siRNA (Supplementary Figures S4A and B). We further examined the potential effect of PDHK4 downregulation on KRAS signalling. We found that the mutant KRAS cells NCI-H460 (Figure 4a) and Calu-6 (supplementary Figure S4C) displayed a significant reduction in ERK1/2 and RSK phosphorylation and total KRAS protein levels following PDHK4 depletion. In contrast, no effect on ERK1/2 and RSK phosphorylation was observed in the KRAS wild-type cell lines, NCI-H1437 (Figure 4a) and cck81 (Supplementary Figure S4C). To study the KRAS activity we performed pull-down assays with the RAS-GTPase protein binding domain of RAF-1 (Raf-RBD) tagged with GST which facilitates the capture and enrichment of the GTP-bound ‘active’ form of KRAS. To avoid confounding results, KRAS activity was assessed at earlier time points following PDHK4 siRNA treatment, where the effect on the total KRAS protein levels was not profound. The pool of active KRAS was greatly reduced in mutant KRAS cells compared to wild-type cells following PDHK4 depletion (Figure 4b). Moreover, the same effect of reducing KRAS signalling and activity was confirmed in the isogenic KRAS mutant HCT116 cells compared to wild-type cells (Figure 4c). Consistent with previous data, no differences in pERK and KRAS activity were observed after treatment with the PDHK1/2 inhibitor AZD7545.

KRAS is modified post-translationally via CAAX prenylation to allow its interaction with the plasma membrane which is required for KRAS activity and signalling pathway activation.^27^ Since PDHK4 regulates lipid metabolism we tested whether PDHK4 depletion could affect KRAS association to the plasma membrane. Confocal microscopy imaging revealed that endogenous RAS protein was predominantly localized at the plasma membrane or other intracellular compartments in mutant KRAS cells, and excluded from the nucleus (Figure 4d). Importantly, PDHK4 knockdown resulted in a re-localization of RAS to the cytoplasm (Figure 4d). Fractionation analysis revealed that while PDHK4 is expressed mainly in the cytoplasm and nucleus, KRAS is expressed predominantly in the cytoplasmic and membrane fractions (Figure 4e, Supplementary Figure S4D). However, most of the active GTP-bound KRAS pool was found in the membrane fraction (plasma membrane, mitochondria, Golgi and/or ER), and excluded from the cytoplasm. Importantly, quantification analysis confirmed a significant reduction of the active KRAS pool in the membrane fraction upon PDHK4 knockdown (Figure 4e, Supplementary Figure S4D). Taken together, these results suggest that PDHK4 is required to maintain the biological activation of mutant KRAS and its association with the cell membrane.

### PDHK4 depletion causes a different metabolic profile in mutant compared to wild-type KRAS cells

**Figure 5:**
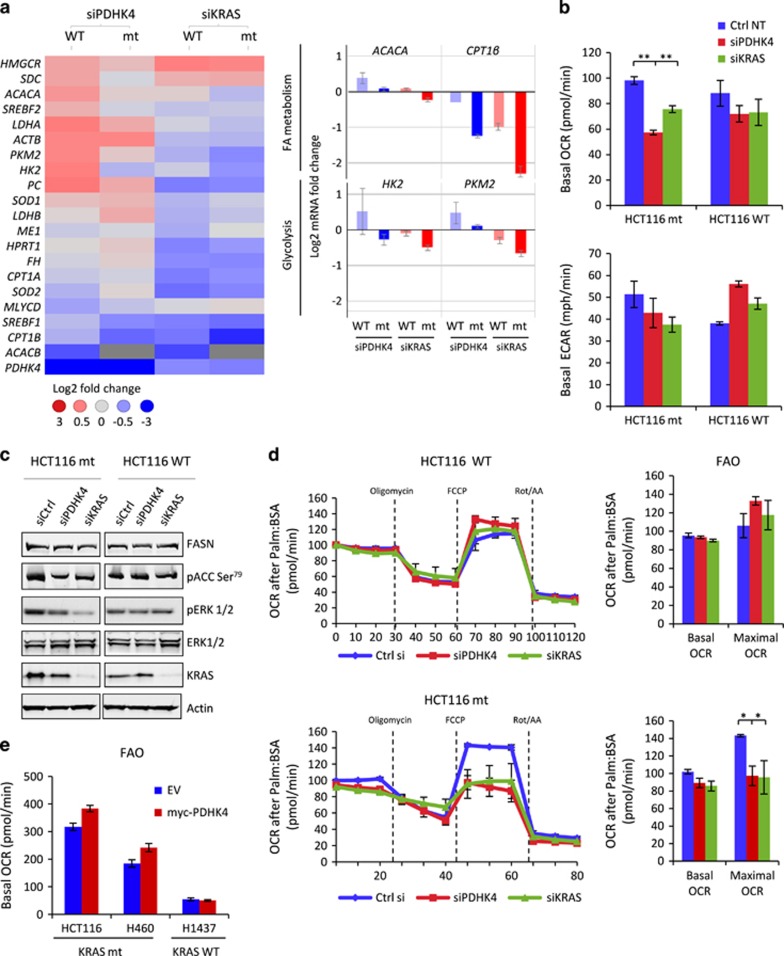
PDHK4 depletion causes opposite metabolic changes in mutant and wild-type KRas cells. (**a**) Fluidigm expression analysis was performed in isogenic HCT116 cells after PDHK4 and KRAS knock-down for 48 h. A heatmap of some of the most relevant genes involved in different metabolic pathways was generated normalizing to TFRC house-keeping gene and represents the log fold change *C*_*t*_ values *n*=3 as indicated (3 to −3). Bar charts on the right represent some of the metabolic genes downregulated in mutant cells after both KRAS and PDHK4 depletion compared to WT cells. (**b**) Seahorse Bioscience XF96 extracellular flux analyser was used for the real-time determination of metabolism in isogenic HCT116 cells after 48 h of KRAS and PDHK4 siRNA treatment (10 nM). Basal OCR (upper chart) and ECAR (lower chart) rates were quantified by SeaHorse analysis and normalized to protein content after Glucose (10 mM) and glutamine (2 mM) addition 2 h before the assay. (**c**) Western blot analysis in HCT116 cells as indicated show the effect on ACC and ERK phosphorylation, FASN, KRAS and Actin protein levels after 40 h of PDHK4 and KRAS siRNA treatment (10 nM). (**d**) Exogenous fatty acid utilization was analysed by the SeaHorse analyser XF96 in isogenic HCT116 cells after 48 h of PDHK4 and KRAS knockdown, and 45 min after addition of 30 μM of BSA-palmitate as the only substrate. Oligomycin (2.5 μg/ml), FCCP (1.5 μM), rotenone (2 μM) and antimycin A (4 μM) were added at the same time point for each experiment. Basal fatty acid oxidation corresponded to the last OCR measurement before Oligomycin addition. Quantification of the maximal FAO rate was determined by the difference of the OCR rate after FCCP addition and the OCR rate after Rotenone/antimycin (Rot/AA) addition. (**e**) Cell lines indicated were transfected for 24 h with the empty vector (EV) control and a full length PDHK4 construct and FAO SeaHorse analysis was performed as in (**d**). All graphs show the mean±s.e.m (*n*=3) and *P*-values were calculated according to *t*-student scores **P<0.05 two-sided paired *t*-test. BSA, bovine serum albumin

PDHK4 mRNA has been shown to be upregulated in response to glucose deprivation and fatty acid supplementation; manifestations which occur in patients with insulin resistance, obesity and type 2 diabetes.^28^ In order to determine whether the observed phenotypic effect on cell growth in mutant KRAS cells after PDHK4 depletion was conferred by a metabolic switch, we first performed a targeted fluidigm expression analysis of the most relevant genes involved in different metabolic pathways in the isogenic HCT116 cells. Interestingly, we found that some of the genes involved in lipid biosynthesis (*SCD, SERBF2, ACACA* and *HMGCR*) and glycolysis (*PKM2, LDHA* or *HK2*) were significantly upregulated in wild-type cells after PDHK4 depletion, compared to mutant KRAS cells (Figure 5a, left panel). However, PDHK4 knockdown did not modulate genes linked to TCA cycle or oxidative stress such as *ME1*, *FH*, *SOD1* and 2. Furthermore, statistical analysis shows some of the genes such as *CPT1β* (involved in the fatty acid oxidation (FAO)), *PC* and *PKM2* (involved in glycolysis) were significantly downregulated in mutant cells compared to wild type after both PDHK4 and KRAS downregulation (Figure 5a). As previously shown, PDHK4 knockdown does not affect the PDH phosphorylation status in KRAS mutant cells (Figure 1d, Supplementary Figure S3C). However, the basal OCR was found to be significantly lower in these cells after PDHK4 and KRAS downregulation (Figure 5b Supplementary Figures S2A, S5A and B). Moreover, a slight reduction in basal extracellular acidification rate (ECAR) was also observed in mutant cells, but KRAS wild-type cells exhibited a slightly higher ECAR levels after PDHK4 knockdown, which would be consistent with the glycolytic gene upregulation observed (Figure 5b Supplementary Figure S5B). Taken together these data support the hypothesis that the metabolic effect of PDHK4 knockdown in KRAS mutant cells is driven through a different mechanism other than through PDH.

PDHK4 has also been implicated in an increase in mitochondrial fatty acid β-oxidation through inactivation of acetyl-CoA carboxylase (ACC) by AMP-activated protein kinase phosphorylation.^29^ Moreover, mutant KRAS expressing tumours also rely on fatty acid metabolism to maintain TCA cycle activity, proliferation and survival.^30, 31^ To further explore the metabolic effects of PDHK4 and KRAS knockdown on lipid metabolism, we analysed ACC and FASN by western blot in the isogenic cells (Figure 5c). Both PDHK4 and KRAS knockdown caused a modest reduction of ACC phosphorylation levels in KRAS mutant cells, but not wild-type cells and no changes were observed on FASN protein levels in either cell line (Figure 5c and Supplementary Figure S5C). To confirm whether this reduction in ACC phosphorylation was functionally relevant, we performed an FAO SeaHorse analysis by measuring the OCR after palmitate addition as only fuel. Interestingly, the reduction in ACC phosphorylation and the *CPT1β* downregulation seen previously (Figure 5a) were consistent with a decrease in basal and maximal FAO in the KRAS mutant cells after PDHK4 and KRAS depletion, but not in the wild-type cells (Figure 5d). Moreover, basal FAO levels were slightly increased by expressing a full-length PDHK4 construct in mutant KRAS cells alone compared to empty-vector construct control cells (Figure 5e). These results indicate a similar reduction in glucose and FAO after PDHK4 and KRAS downregulation in mutant KRAS cells, suggesting that these metabolic changes could be driven as a consequence of PDHK4-dependent downregulation of KRAS function.

### Stable PDHK4 expression enhances mutant KRAS activity in the plasma membrane, inducing *in vitro* cell growth in 3D cultures and *in vivo* tumour growth

**Figure 6:**
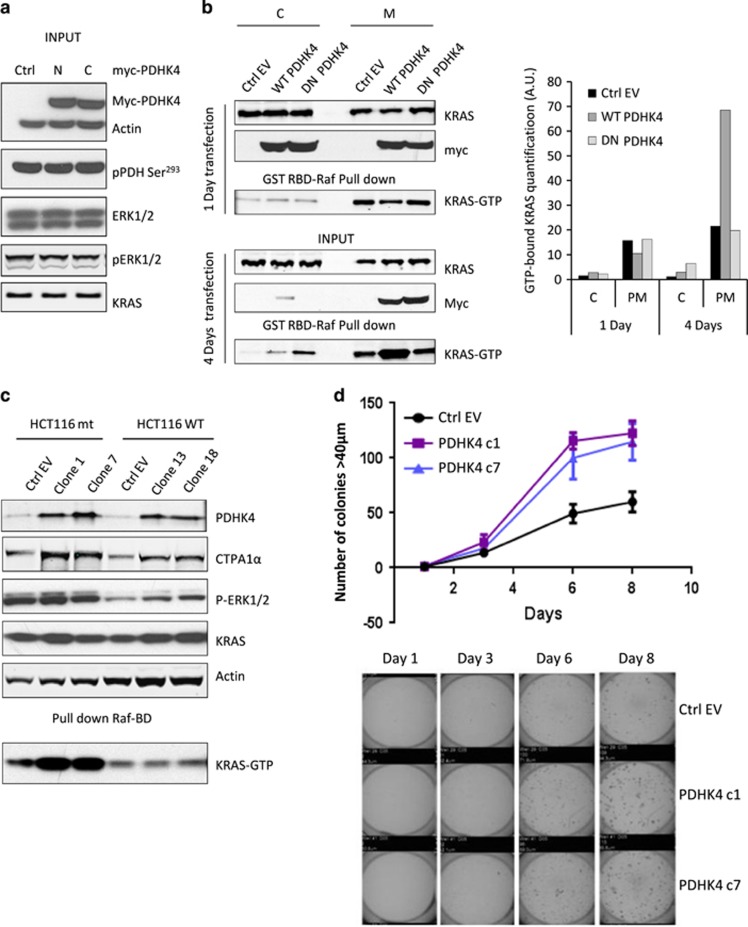
Stable PDHK4 expression enhances mutant KRAS activity in the plasma membrane and induces *in* vitro cell growth in 3D cultures. (**a**) HCT-116 cells were transiently transfected for 24 h with two full length constructs of PDHK4 (1 μg), either N- or C-terminally labelled with a myc-tag (3 μg). Western blotting was carried out to determine which construct gave the best expression levels. (**b**) HCT116 cells were transfected with the PDHK4 full-length (1 μg) and dominant negative (DN, 3 μg) C-terminal myc tag constructs for 1 and 4 days followed by cell fractionation. Each fraction was subjected to immunoblot and KRAS GTPase pull-down assay to detect activity. Chart represents the quantification of GTP-bound KRAS in the cytoplasmic and membrane fractions normalized to total KRAS levels. (**c**) PDHK4 stable clones were generated with the full-length PDHK4 C-terminally myc-tag construct in HCT116 KRAS WT (clones 13 and 18) and mutant (clones 1 and 7). Protein extracts were subjected to KRAS GTPase pull-down assays and expression levels of indicated proteins including KRAS-GTP bound were verified by western blot. (**d**) KRAS mutant PDHK4 clones 1, 7 and empty vector (EV) were suspended into 0.3% agarose and plated on a 1% agar bottom layer. Colonies>40 μm were counted using a Gelcount scanner every 2 days for 2 weeks. Error bars represent the ±s.e.m of replicates (*n*=5).

**Figure 7:**
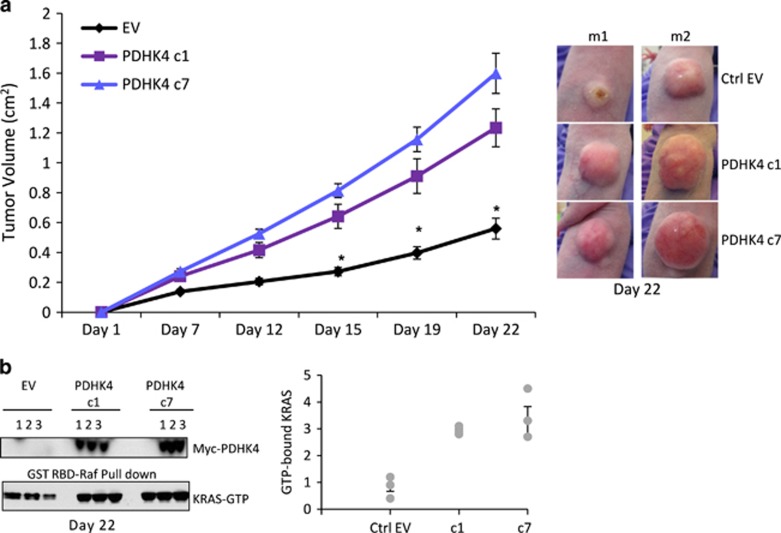
PDHK4 expression promotes mutant KRAS tumour growth *in vivo*. (**a**) Nude mice were injected subcutaneously with 1 × 10^7^ of the indicated HCT116 KRAS mutant clones (EV, clones 1 and 7) and tumour growth was monitored twice weekly by bilateral calliper measurements for 22 days after injection. Mice were randomized into vehicle or treatment groups with approximate mean start size of 0.2–0.4 cm^3^. Error bars represent the ±s.e.m of individual mice (*n*=10) used per condition. *P*-values were calculated using one-tailed unpaired Student *t* test for comparisons of the groups. *P–values <0.0001. (**b**) The tumours were analysed 22 days post-injection for KRAS activity and myc-PDHK4 total protein expression by western blot analysis. Graph represents quantification of the GTP-bound KRAS of three individual animals and error bars represented as ±s.e.m of replicates (*n*=3).

PDHK4-dependent reduction in KRAS activity highlights an interesting link between these two proteins. Since the PDHK4 expression is not high in these tumour cells, we studied the effect of full-length PDHK4 over-expression on KRAS, pERK and pPDH levels. No changes were seen in the levels of KRAS, pERK or pPDH Ser^293^ after 24 h of transient transfection with N- or C-terminally labelled myc-PDHK4 constructs (Figure 6a). In addition, we generated a C-terminally truncated catalytically inactive variant, mutated in two conserved residues (DW) within the ATP binding pocket of PDHK4 (D394A and W395A) that are essential for the catalytic activity and the correct folding of the enzyme.^32^ After 1 day of transfection, the over-expressed PDHK4 and endogenous KRAS were found in the cytoplasmic and membrane fraction. Interestingly, after 4 days of transfection, the PDHK4 full-length protein and the dominant negative PDHK4 protein (DN-PDHK4) appeared to be re-localized exclusively in the membrane fraction. Moreover, expression of full-length wild-type PDHK4, but not the dominant negative, resulted in a significant enhancement of the active KRAS pool in the membrane fraction (Figure 6b). In addition, we generated stable cell lines over-expressing PDHK4 to compare the functional effect of PDHK4 in KRAS mutant and wild-type cells. We found that the expression of PDHK4 in mutant KRAS clones led to a significant enhancement in active mutant KRAS, but no effect was observed when PDHK4 was over-expressed in the wild-type KRAS clones (Figure 6c). These results suggest a direct or indirect effect in KRAS activation when kinase active PDHK4 is stably expressed. We then assessed the impact of PDHK4 over-expression in 3D cultures that are characteristic of KRAS cellular transformation and uncontrolled cell growth. We found that mutant KRAS clones stably expressing PDHK4 displayed a significant increase in colony formation ability in soft agar assays, compared to cells expressing empty vector (Figure 6d). We finally explored whether this effect on cell growth in the *in vitro* 3D cultures and KRAS activation could be translated to an increase in xenograft tumour growth *in vivo*. Importantly, *in vivo* tumour growth 22 days post-implant was significantly greater in tumours derived from mutant KRAS -PDHK4 stable clones 1 and 7 than in the empty vector clone (Figure 7a and Supplementary Figure S5E). Protein expression of myc-PDHK4 was also confirmed by western blot analysis of xenograft extracts 22 days post-implant and pull-down assays also showed an increase in KRAS activity in the PDHK4 stable xenograft compared to the control tumours (Figure 7b). Taken together, these data strongly support that PDHK4 regulates, directly or indirectly, KRAS activity and KRAS-dependent cell growth, and highlights a novel pathway regulating KRAS-driven tumorigenesis.

## Discussion

Over recent years there has been a significant increase in effort in developing effective therapeutic strategies to target KRAS mutant tumours either through direct targeting of KRAS or indirect targeting of the RAS-MEK-ERK signalling pathway.^33^ Direct pharmacological inhibition of RAS has proved challenging due to the high affinity of RAS for GTP. Moreover, the intensive efforts to develop inhibitors of RAS post-translational modifications such as FTase inhibitors have failed in the past due to compensatory activity of the geranyl modifications.^34, 35^ In contrast, clinical studies have demonstrated improved efficacy by inhibiting the RAS effector signalling pathways and the next generation of RAF, MEK and ERK inhibitors have entered clinical trials demonstrating promising clinical activity in metastatic melanoma and other tumour types. However, toxicity issues, variable responses rates and development of resistance due to the activation of other kinases are limiting the efficacy and the clinical progression of these compounds as monotherapy treatment.^36^ Most recently, work revealing the involvement of KRAS in driving the metabolic phenotype of tumours provides a unique opportunity to search for alternative metabolic approaches to target KRAS mutant tumours.^37^

Pharmacological inhibition, or decreased expression of the PDHK isoforms, has been reported to reduce xenograft tumour growth of a variety of tumour cell line models.^14, 38, 39^ Some studies have evaluated the role of PDHK1 in tumorigenesis, for example, demonstrating that PDHK1 plays an important role in oncogene-induced senescence in BRAF^V600E^ melanomas and has been demonstrated to be required for metabolic adaptation to nutrient deprivation and hypoxia in a metastatic breast cancer cells.^22, 40^ In this study we have identified that the proliferation of KRAS mutant cancer cells are dependent on a PDHK4, which represents a potentially druggable kinase. When extended to a larger panel of cell lines we observed enhanced sensitivity to PDHK4 depletion in KRAS mutant versus wild-type tumour cells. Recently, PDHK4 has been shown to promote tumorigenesis through mTORC1 signalling^41^ and knockdown of PDHK4 has been shown inhibit colorectal tumour growth.^42^ In addition, PDHK4 expression is positively correlated with drug resistance in colon cancer cells and induced by 5-FU.^42, 43^ In contrast, other studies have proposed a specific suppressive role on proliferation after PDHK4 upregulation in MCF-10A mammary epithelial cells and lung tumour cells following PPARγ upregulation.^44, 45^ Further work report that PDHK4 expression could be regulated by different microRNAs. While PDHK4 is negatively regulated by miR-211 leading to an inhibition of invasion in melanomas,^46^ another study has demonstrated that upregulation of miR-182 in two lung cell lines is correlated with PDHK4 downregulation and cell growth promotion.^47^ These opposing roles of PDHK4 in tumorigenesis may depend on tumour’s tissue of origin and the complexity of the genetic background.

In this study, most of the lung and colorectal KRAS mutant cell models displayed a marked dependence of cell growth on PDHK4 expression and this dependence was not evident in wild-type cells. In this KRAS mutant context, PDHK4 downregulation did not affect the PDH phosphorylation levels (Ser^293^ or Ser^300^), suggesting that PDHK4 could play a previously un-appreciated role in mutant KRAS cancer models. Importantly, although PDHK4 did not modulate KRAS mRNA levels, a strong reduction in mutant KRAS activity, a change in its cellular localization and an inhibition of RAS downstream signalling was observed.

Recently, small molecule inhibitors of phosphodiesterase delta have been shown to regulate KRAS trafficking to membrane compartments, modifying KRAS localization and activation.^48^ It has been shown that RAS-transformed cells take up exogenous lipids to provide cells with fatty acids, thereby decreasing the need for *de novo* synthesis,^31^ indicating that KRAS activation could be affected by the availability of exogenous fatty acids and/or the ability to synthesize *de novo* fatty acids within the cells.^30^ Interestingly, the biological role of PDHK4 in normal tissues is linked to insulin resistance in diabetes and fatty acid metabolism. Indeed, upregulation of PDHK4 in normal tissues by the glycerol biogenesis pathway and the farnesoyd X receptor leads to a decrease in glucose oxidation and an increase in FAO.^49, 50^ Moreover, under starvation, PDHK4-deficient mice showed a greater accumulation of non-esterified lipids in the blood and less FAO compared to the wild-type mice.^20^ Interestingly, our work revealed that both PDHK4 and KRAS knockdown displayed a similar metabolic phenotype in KRAS mutant cells, consisting of a reduction in OCR after glucose and FAO, which was not observed in wild-type cells. This significant reduction in FAO might be a consequence of lowering the lipid uptake that would force an increase of *de novo* fatty acid synthesis after KRAS depletion. Here, we clearly demonstrate that PDHK4 knockdown has an effect on KRAS localization and activity. This may be due to a direct or indirect effect on lipid metabolism that impacts on KRAS membrane localization (where KRAS needs to be anchored to remain active), resulting in a reduction in KRAS activation.

In support of this hypothesis, cells stably over-expressing PDHK4 exhibited an increase in KRAS activity and 3D cell growth *in vitro* and *in vivo*, suggesting a role for PDHK4 in the growth of KRAS mutant tumours. Whether this is a direct, or an indirect effect on KRAS activation, our findings provide evidence of a previously unappreciated mechanistic link between the metabolic kinase PDHK4 and mutant KRAS biological function. To date, the metabolic significance of PDHK4 in cancer development is still unclear but this study proposes a new role of PDHK4 in regulating mutant KRAS activation and tumour promotion.

Further studies are required to explore this mechanistic link in other KRAS-dependent tumour types such as pancreatic cancer to understand the extent of the potential therapeutic opportunity.

## Materials and methods

### Cell culture and transfections

The isogenic HCT-116 cell line pair were obtained from Horizon Discovery (Cambridge, UK). The mutant allelle in the parental cells (KRAS mt) was replaced by a wild-type KRAS allele to form an HCT-116 cell line that is KRAS wild type (KRAS WT). Other cells used were obtained from the ATCC and authenticated at AstraZeneca global cell bank using DNA fingerprinting short-tandem repeat assays. All cell lines were grown in RPMI 1640 media+10% fetal calf serum+1 mM glutamine at 37 °C and 5% carbon dioxide. Single and SmartPOOL siRNAs (Dharmacon, Lafayette, CO, USA) were re-suspended in water to 10 μM and reverse transfected by adding 10 nM final concentration premixed with Lipofectamine RNAiMAX (Invitrogen, Carlsbad, CA, USA) in OPTI-MEM (Gibco, Waltham, MA, USA). shRNA and over-expression constructs were transiently transfected using Genejuice (Merck Biosciences, Billerica, MA, USA). PDHK4 full-length constructs with N- and C-terminal myc-tags were generated by GeneArt using pcDNA3-myc vector (Invitrogen) and PDHK4 accession number NM_013743. A variant classified as dominant negative was generated by mutation of PDHK4 in two conserved residues (DW) within the ATP binding pocket (D394A and W395A). pGIPZ MicroRNA-adapted shRNA were obtained by Dharmacon for specific inducible PDHK4 knockdown (Catalogue number RMM4431-99941091, RMM4431-99943511, RMM4431-99946611, RMM4431-99947383).

AZD7545 was synthesized by AstraZeneca (Macclesfield, UK) and re-suspended in dimethyl sulfoxide to 10 mM. Dichloroacetate and Doxycycline was obtained from Sigma (St Louis, MO, USA) and re-suspended in dimethyl sulfoxide to 1 M and 10 mg/ml respectively. Geniticin antibiotic was obtain from Gibco and at a concentration of 200 mg/ml re-suspended in pure water.

### Cell growth and cytotoxicity assays

#### Sytox green assay

Cells were plated into a 96-well black clear-bottomed plate (Costar, Washington, DC, USA). The following day cells were transfected or treated with indicated compounds and cultivated for 3–5 days. A day 0 plate was set up to define the baseline cell number; this plate was assayed at the time of treatment. Dead cells were detected by adding 5 μl of 2 μmol/l Sytox Green (Life Technologies, Carlsbad, CA, USA) in Tris-buffered saline (TBS)/5 mmol/l ethilenediaminetetraacetic acid (EDTA) to each well of the 96-well plates followed by incubation for 1 h at room temperature. Cells that had taken up the dye were counted on the Acumen automated imaging system. Subsequently, 10 μl of 0.25% saponin in TBS/5 mM EDTA was added to each well, incubated at room temperature for 16 h and re-read using the Acumen for a total cell number read-out.

#### Sulphorhodamine B colorimetric assay

Homogeneous plating and cell count was assessed by fixing the cells with 10% trichloroacetic acid for 1 h at 4 °C and then staining the fixed cells with 0.47% solution of Sulforhodamine B (Sigma). Plates were washed five times with 1% acetic acid solution and dried for 20 min at room temperature. The protein-bound dye was then dissolved in 10 mM Tris base solution for optical density determination at 510 nm using a microplate reader. The results are linear over a 20-fold range of cell numbers.

### Immuno-fluorescence and microscopy

Cells were grown on glass cover slips and washed with phosphate-buffered saline (PBS) after siRNA treatment. Next, cells were fixed in 4% paraformaldehyde and permeabilized with 0.5% Triton X-100 in PBS. Cells were washed with PBS and blocked in 1% bovine serum albumin-PBS. Pan-Ras and myc (9E10) antibodies (Millipore, Billerica, MA, USA) were used at a 1:100 dilution and Alexa 488-conjugated or Alexa 594-conjugated secondary antibodies (Invitrogen) were used for visualization. 4',6-diamidino-2-phenylindole (Sigma) 0.5 mg/ml was used as a nuclear stain. Confocal analysis was performed using the Leica SP5 Multi-photon Confocal Microscope equipped with UV Diode (405 nm), argon (458, 476, 488 and 514 nm), HeNe (543 nm) and a HeNe (633 nm) lasers.

### mRNA gene expression analysis

RNA was prepared using an extraction column according to the manufacturer’s instructions (Qiagen, Hilden, Germany). Complementary DNA was generated using first-strand cDNA kit (Invitrogen) according to the manufacturer’s instructions on 2 μg DNAse-treated total RNA. Quantitative RT–PCR (qRT–PCR) analysis was performed with 2 μl of a 100 × dilution of the cDNA, 2 μl of each primer 20 μM and carried out with the DyNAmo SYBR Green two-step qRT–PCR kit (Thermo Scientific, Waltham, MA, USA). Accumulation of fluorescent products was monitored by real-time PCR using a Chromo4 reader (Bio-Rad, Hercules, CA, USA), melting curves determined and was analysed with the Opticon Monitor3 software. The relative quantification of gene expression was performed using the ΔΔC_T_ method, with normalization of the target gene to the ribosomal control gene glyceraldehyde 3-phosphate dehydrogenase.

### Gene profiling and analysis

RNA was prepared using an RNeasy extraction kit according to the manufacturer’s instructions (Qiagen). Quantitative RT–PCR (qRT–PCR) analysis was performed with 2 μl of a 1:100 dilution of the cDNA, 2 μl of each primer (20 μM) and carried out with the DyNAmo SYBR Green two-step qRT–PCR kit (Thermo Scientific). Accumulation of fluorescent products was monitored by real-time PCR using a Chromo4 reader (Bio-Rad), melting curves determined and data analysed with the Opticon Monitor3 software. Targeted gene expression was performed using a 48 × 48 Fluidigm dynamic array and ABI primers (Thermo Scientific). The Fluidigm Array was then primed and loaded on an IFC Controller and qRT–PCR experiments run on the Biomark System. Data were collected and analysed using the Fluidigm Real-Time PCR Analysis software, generating Ct values.

### Protein extraction, immuno-precipitation and immuno-blots

Cells were washed with cold PBS and lysed directly on the plate in cold NP-40 lysis buffer (50 mM Tris-HCl pH=7, 150 mM NaCl, 1 mM EDTA and 1% NP-40 supplemented with a complete protease and phosphatase inhibitor cocktail). Western blot analysis was carried out using the following specific antibodies: PDHK4 (Novus, Littleton, CO, USA), myc 9E10, Pan-Ras, PDHα, P-PDH s293 (Millipore), KRAS (LS Bio, Seattle, WA, USA), pERK 1/2, Cleaved-Caspase3, Na/K-ATPase, p-Acc s79, Histone-3A (CST, Danvers, MA, USA), pRSK, FASN (BD Biosciences, Franklin Lakes, NJ, USA), β-Actin, LDHB, Vinculin (Sigma), NRAS (SantaCruz, Dallas, TX, USA) and CPT1α (Abcam, Cambridge, UK). Intensity values were quantified with Image Studio software 2.0 (LICOR Bioscience, Lincoln, NE, USA).

### 3D soft agar cell growth assay

Colony formation assays were performed in 96-well plates. Tumour cells (1000–2000 per well) were suspended in 0.3% agarose, RPMI 1640, 10% fetal calf serum and placed onto a 1% agarose, RPMI 1640, 10% fetal calf serum layer. Once set at room temperature, 50 μl of media was added on top of the agar and plates incubated at 37 °C and 5% CO_2_. Colony formation was measured every other day for 14 days by scanning and counting colonies greater than 40 μm with a Gelcount scanner (Oxford Optronix, Oxford, UK).

### Measurement of OCR and ECAR

Basal and maximal OCR and ECAR were measured using the SeaHorse XF96 analyser. Following a 2 h starvation, cells were supplemented with 2.5 mM of glucose and 1 mM of glutamine in SeaHorse culture media. OCR and ECAR values were normalized to by Sulphorhodamine staining (Invitrogen). To measure FAO, the medium was replaced 45 min before the reading with KHB buffer (111 mM NaCl, 4.7 mM KCl, 1.25 mM CaCl2, 2 mM MgSO4, 1.2 mM NaH2PO4) supplemented with 0.5 mM carnitine, and 5 mM HEPES, adjusted to pH7.4 at 37 °C. No other fuels such as glucose, glutamine or pyruvate were added to the starvation media, so only bovine serum albumin-palmitate was included as a control. Plates were incubated 30 min at 37 °C before the addition of 30 μl of palmitate:bovine serum albumin substrate and microplate was read into the XF96 Analyser (Agilent, Santa Clara, CA USA).

### *In vivo* tumour growth studies

All studies involving animals in the UK were conducted in accordance with UK Home Office legislation, the Animal Scientific Procedures Act 1986 (ASPA) and AstraZeneca Global Bioethics policy. All experimental work is outlined in project license 40/3483, which has gone through the AstraZeneca Ethical Review Process. HCT116 cells EV and PDHK4 clones were cultured in complete RPMI (Invitrogen). Approximately 1 × 10^7^ cells were implanted subcutaneously in a total volume of 0.1 ml/mouse. HCT116 xenografts were established in female nude mice. All mice were older than 6 weeks at the time of cell implant. Tumor growth was monitored twice weekly by bilateral calliper measurements, tumour volume was calculated with approximate mean start size of 0.2–0.4 cm^3^. Tumour growth inhibition from start was assessed by comparison of the mean change in tumour volume for the EV group and PDHK4 clones.

### Statistical analysis

All *in vitro* data are presented as the standard error of the mean of three replicates. Gene expression calculations and statistical analysis were performed in Jmp12.0.1, and data represented in TIBCO Spotfire 6.5.2. Ct values were normalized to TFRC housekeeping genes and treatments were normalized to untreated dimethyl sulfoxide controls to calculate fold change in gene expression. JMP software was used to calculate *P*-values. (*P*<0.05 two-sided paired *t*-test). *In vivo* data were analysed were presented as s.e.m. (*n*=5 and 10) using one-tailed unpaired Student *t*-test for comparisons of the groups. *P*-values <0.001 were considered statistically significant.
