# Supplementary figures and images for: Pyruvate dehydrogenase kinase 4 exhibits a novel role in the activation of mutant KRAS, regulating cell growth in lung and colorectal tumour cells
Source: Oncogene. 2017 Jul 10;36(44):6164–76. doi: 10.1038/onc.2017.224 (PMC5671936; doi:10.1038/onc.2017.224)

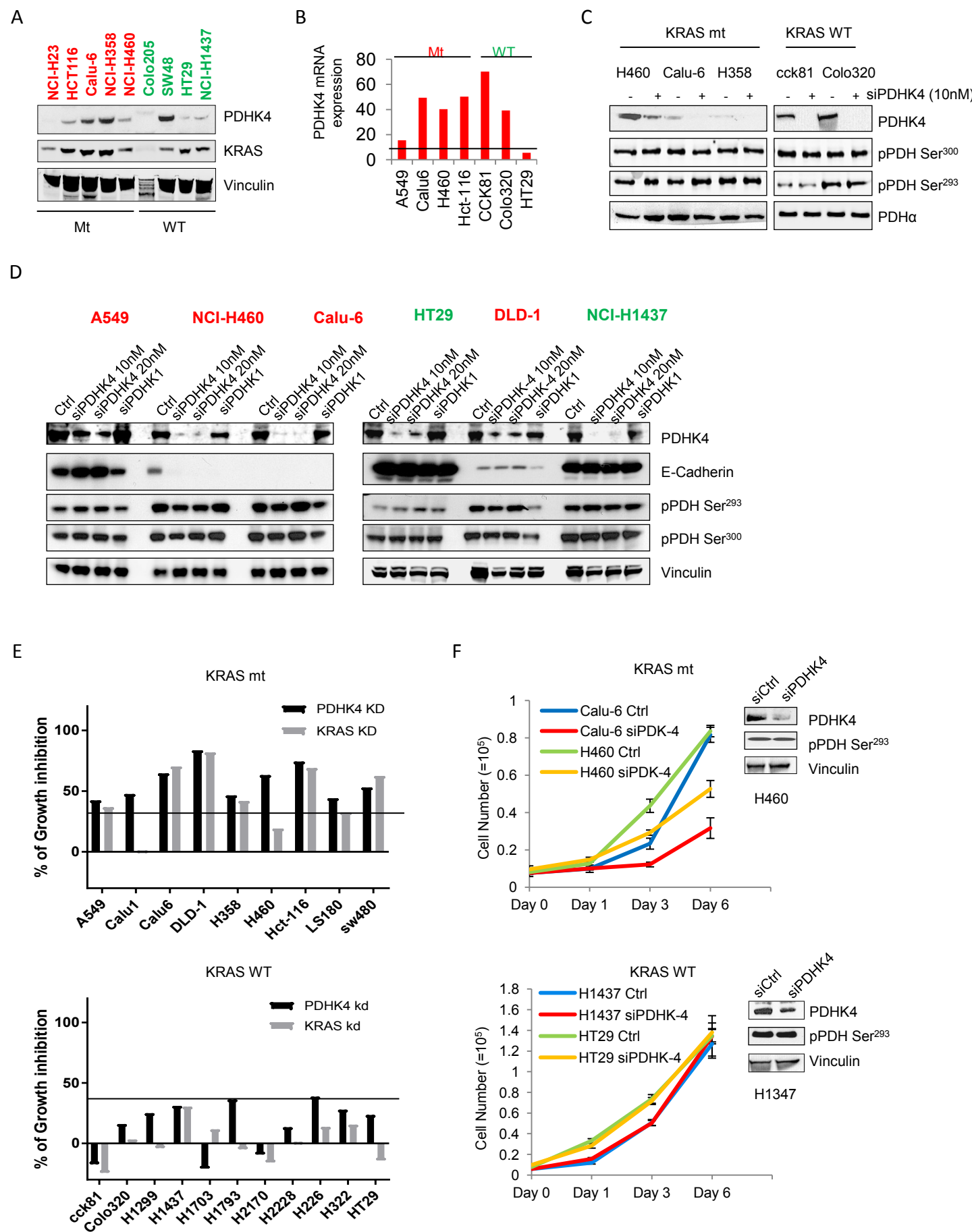

Supplementary Figure 1

A

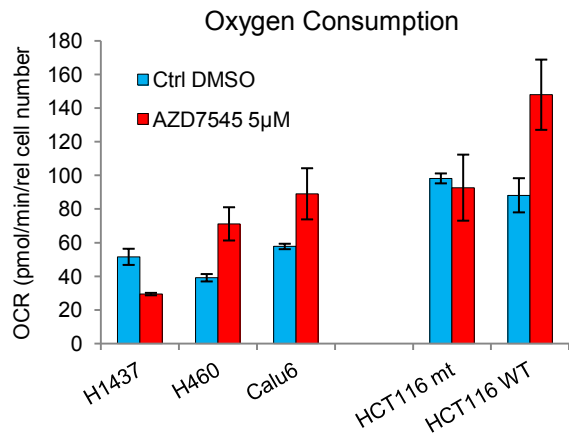

B

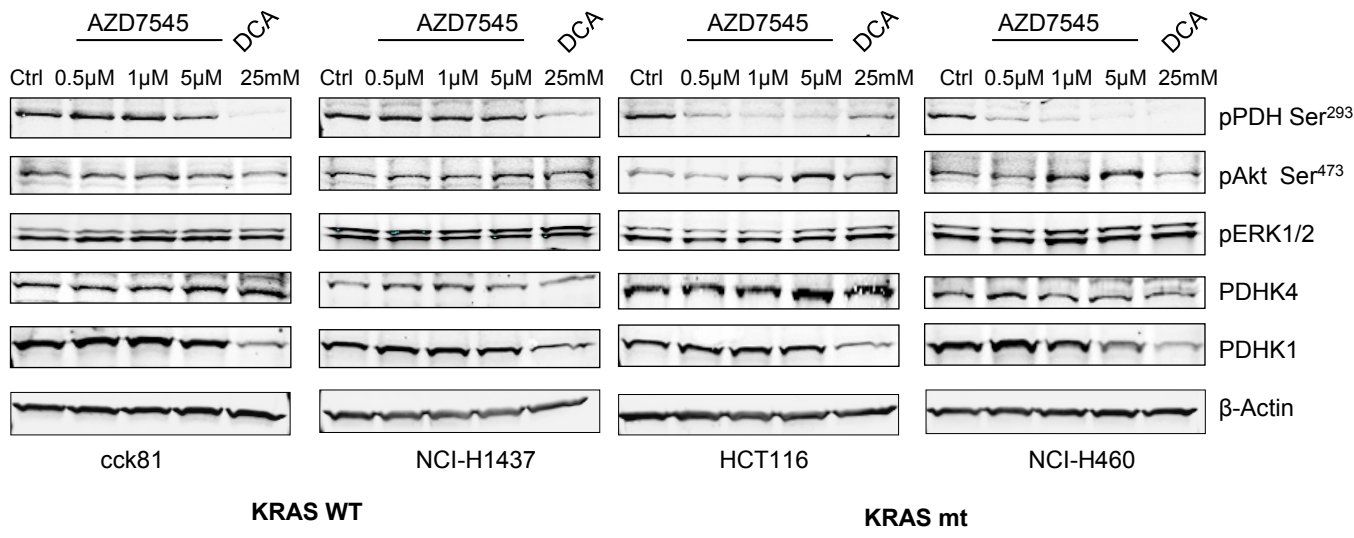

A

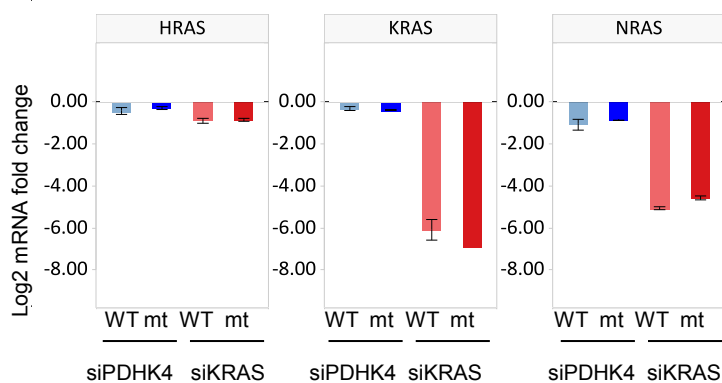

B

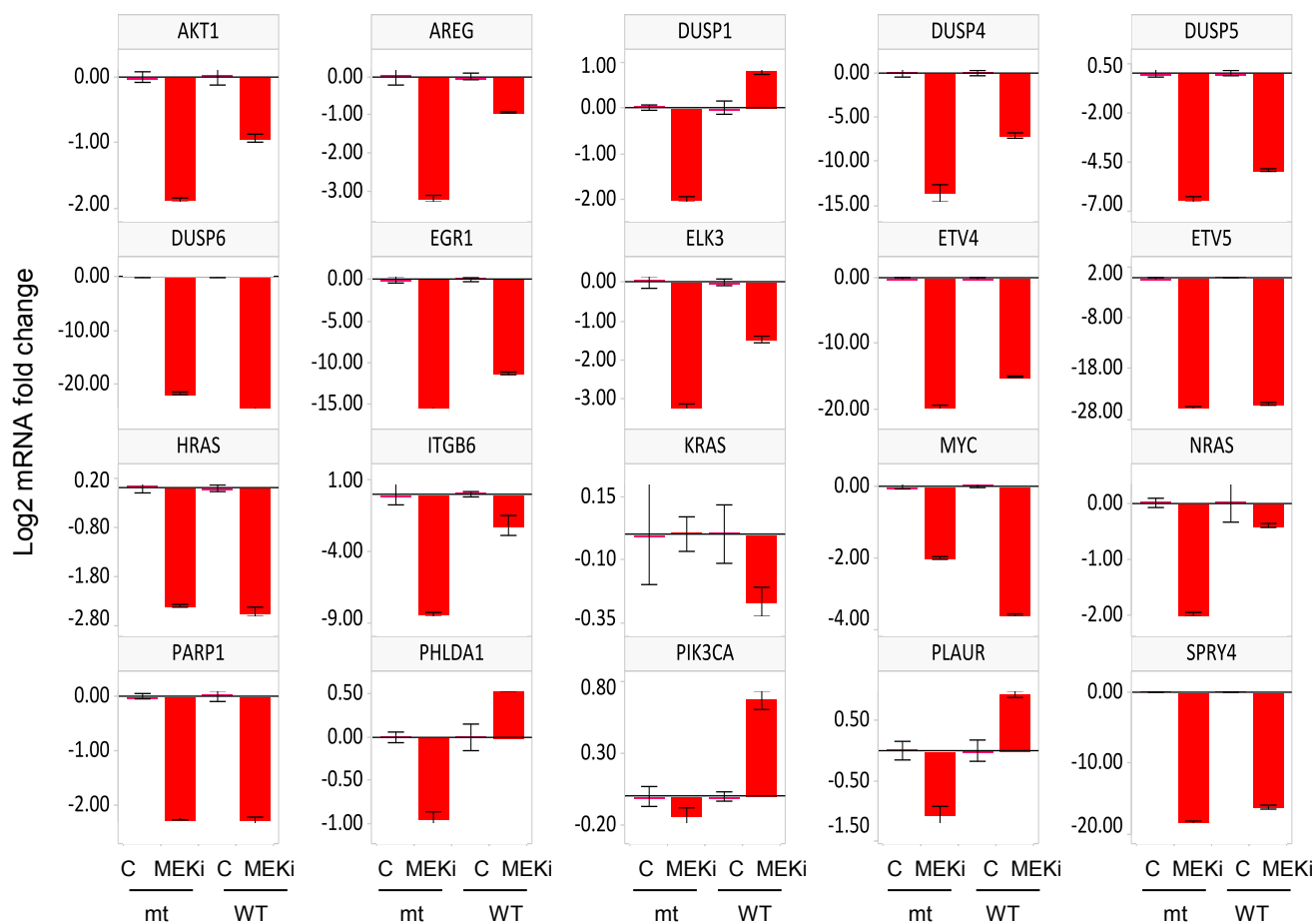

A

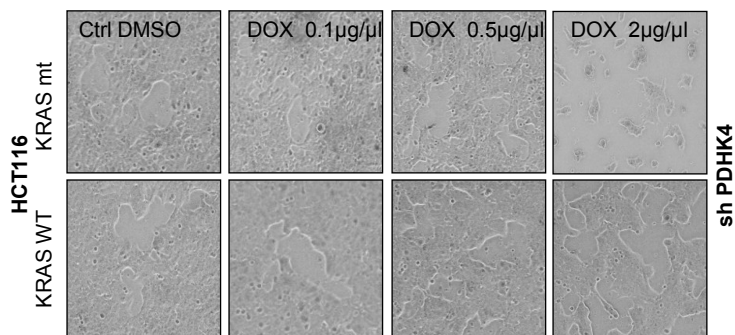

B

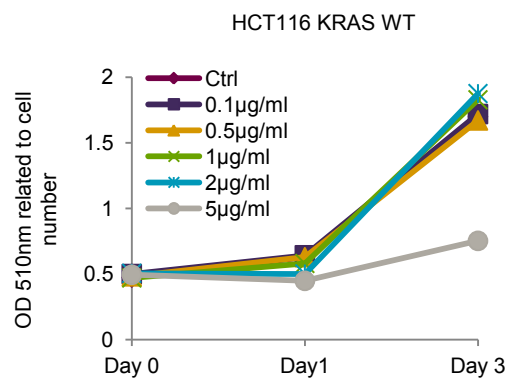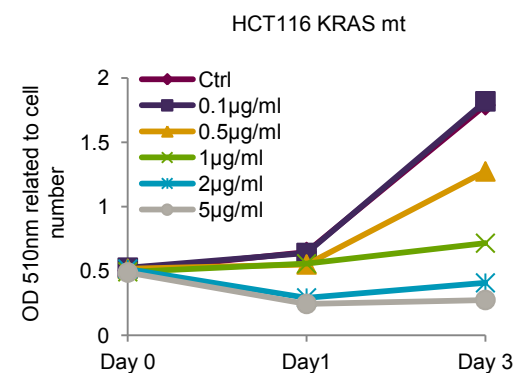

C

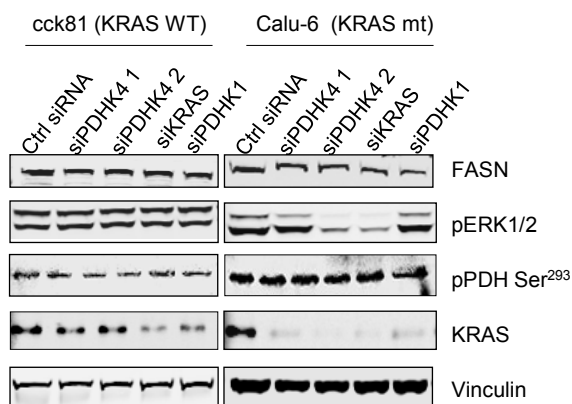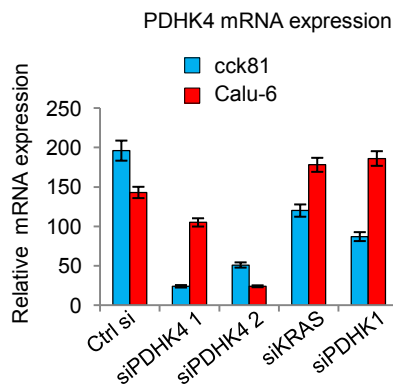

D

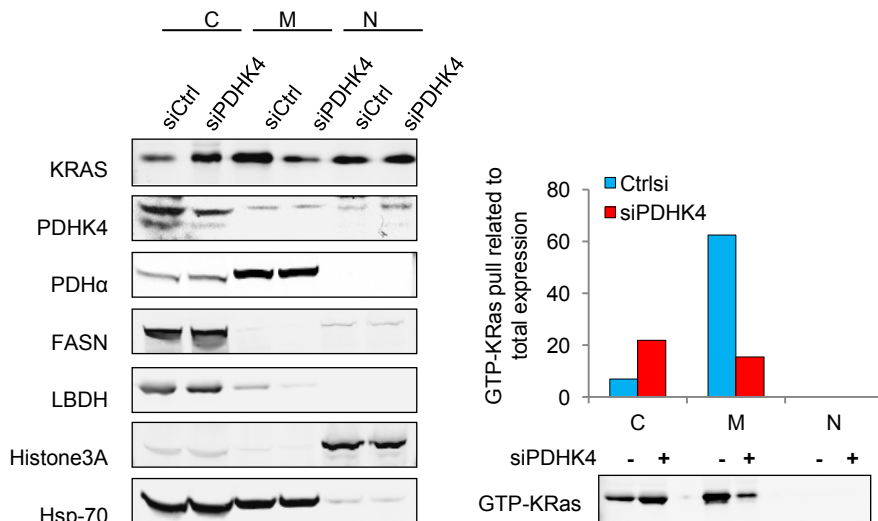

Supplementary Figure 4

A

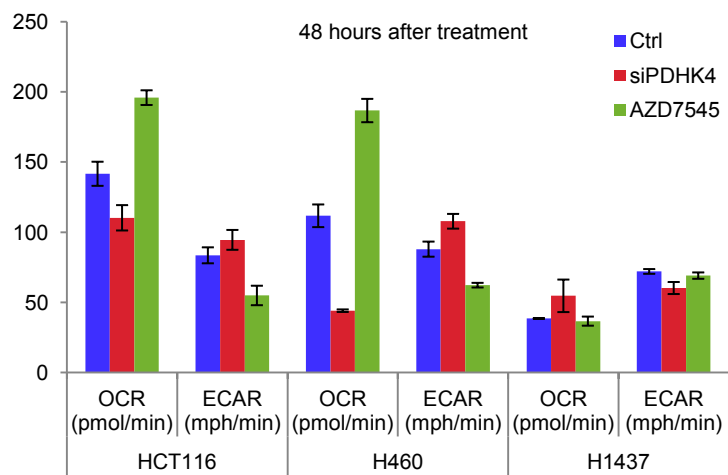

C

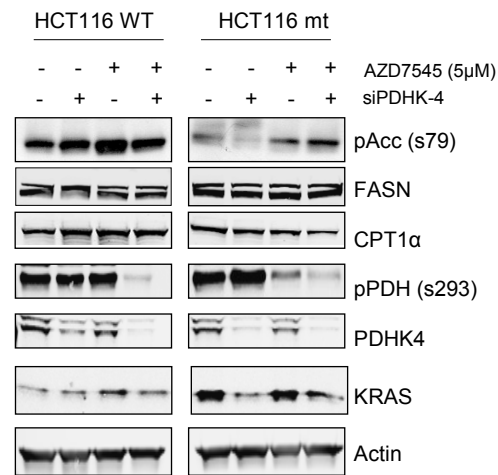

B

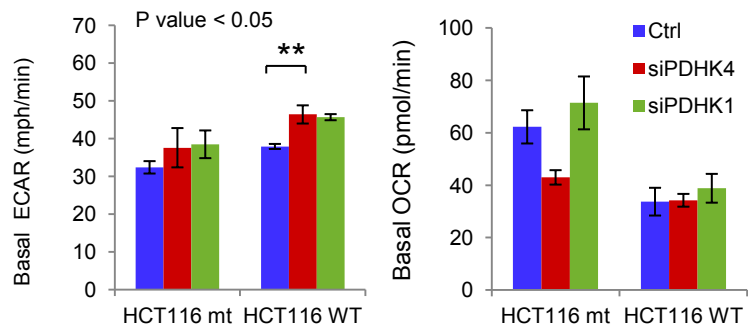

D

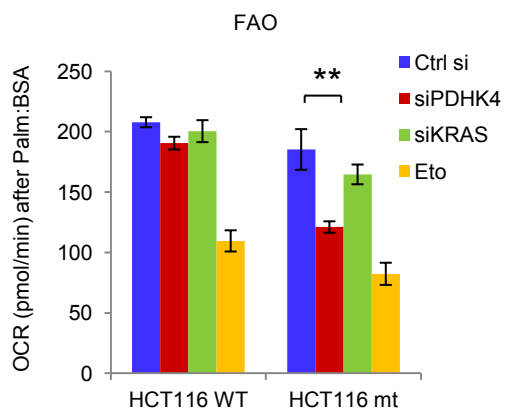

E

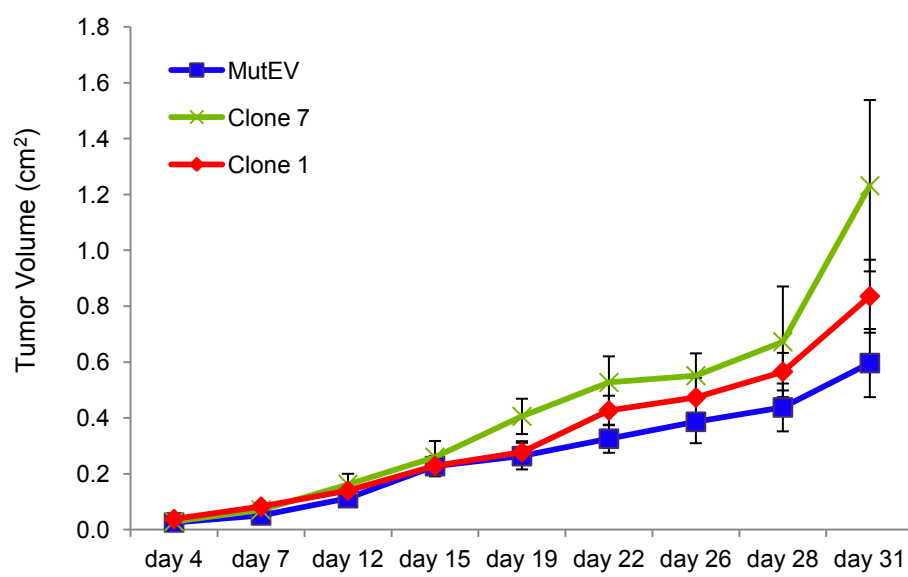

Supplement: Supplementary Figures [file onc2017224x1.pdf]
