# Supplementary material for: Pyruvate dehydrogenase kinase 4 exhibits a novel role in the activation of mutant KRAS, regulating cell growth in lung and colorectal tumour cells
Source: Oncogene. 2017 Jul 10;36(44):6164–76. doi: 10.1038/onc.2017.224 (PMC5671936; doi:10.1038/onc.2017.224)
Supplement: Supplementary Figure Legends [file onc2017224x2.docx]

**SUPPLEMENTARY FIGURE LEGENDS**

**Supplementary Figure S1 a)** KRAS and PDHK4 protein expression levels were tested in mutant and wild-type KRAS cells. **b)** PDHK4 expression levels were assessed by qRT-PCR and relative *C_t_* values were normalised to GAPDH expression. **c,d)** Wild-type and mutant KRAS cells were transfected for 48 hours with PDHK4 and PDHK1 siRNA as indicated and total protein expression levels and PDH Ser^293^ and Ser^300^ phosphorylation were analysed by immunoblot. **e)** PDHK4 and KRAS knock-down was performed in KRAS mutant and wild-type cells and the effect on the cell growth was analysed by Sytox green cell count after 72 hours and compared to the control siRNA. Percentage growth inhibition values were represented in the indicated wild-type and mutant cell lines. **f)** Effect of PDHK4 down-regulation in lung wt (H1437 and HT29) and mutant KRAS (Calu-6 and H460) cell lines at indicated times. Cells were harvested after 3 days of PDHK4 knock-down (10 nM) for western blot analysis and protein content was normalised by BCA assay.

**Supplementary Figure S2. a)** Basal oxygen consumption rates (OCR) were measured followed Glucose (10 nM) and glutamine (2 nM) addition with the SearHorse XF96 analyser in the indicated cell lines after 24 hours of treatment with 5 μM of AZD7545. **b)** KRAS wild-type and mutant cells were treated with indicated concentrations of AZD7545 and the Pan PDHK inhibitor DCA. Cells were harvested after 24 hours and protein expression analysis was performed by western blotting, using the indicated antibodies**.**

**Supplementary Figure S3**. **a)** Analysis of Ras isoform expression by Fluidigm dynamic array used in Fig 3C in WT and mutant KRAS HCT116 cells after PDHK4 and KRAS knock-down **b)** Gene expression analysis of selected genes from MEK signature was performed in isogenic HCT116 cells after treatment with the MEK inhibitor AZD6244 (MEKi; 500 nM) for 24 hours. Values represent the changes in gene expression as log2 fold change

**Supplementary Figure S4 a,b)** Isogenic HCT116 cells were transfected with Doxycycline (Dox) inducible PDHK4 shRNA construct for 24 hours followed by Dox induction for 3 days at the indicated concentrations **a)** Transmission pictures were taken with 40X objective and western blot analysis were performed to validate PDHK4 knock-down (10 nM) and to test KRAS and pERK expression levels. **b)** A measure of relative cell number (OD 510 nm) was determined by sulphorhodamine B assay after 0, 1 and 3 days of Dox induction at indicated concentrations. **c)** Calu-6 and cck81 cells were transfected with the indicated specific siRNAs for 48 hours and protein expression analysis was performed by immunoblot. PDHK4 knock-down was validated in both cell lines measuring mRNA levels by qRT-PCR and *C_t_* values were normalised to GAPDH expression. **d)** Cells were fractionated after 48 hours of PDHK4 knock-down and protein localisation was analysed in the cytoplasmic, membrane and nuclear fraction was assessed by immunoblot with the indicated antibodies.

**Supplementary Figure S5**. **a,b**) Basal OCR and ECAR were measured in the indicated cells by the SeaHorse analyser after treatment with **a)** AZD7545 (5 μM) and PDHK4 siRNA (10 nM) and **b)** PDHK1 (10 nM) and PDHK4 (10 nM) siRNA for 48 hours. **c)** Isogenic HCT116 cells were transfected for 24 hours with PDHK4 siRNA followed by treatment with 5 μM of AZD7545 and protein expression levels were tested by western with the indicated antibodies. **d)** Basal OCR was assessed in the Seachorse XF96 analyser after 48 hours of KRAS and PDHK4 siRNA transfection. Fatty acid oxidation inhibitor Etomoxir at 40 μM and 2 μM BSA:palmitate was added 45 minutes before the first basal measurement was taken. **e)** Nude mice were injected subcutaneously with 1x10^7^ of the indicated HCT116 clones and in vivo tumour growth was measured twice a week for 31 days after injection. Error bars represent the +/- SEM of different mice used per condition (n = 5)
